# Plastome sequencing reveals phylogenetic relationships among *Comastoma* and related taxa (Gentianaceae) from the Qinghai–Tibetan Plateau

**DOI:** 10.1002/ece3.8274

**Published:** 2021-10-28

**Authors:** Yu Zhang, Jingya Yu, Mingze Xia, Xiaofeng Chi, Gulzar Khan, Shilong Chen, Faqi Zhang

**Affiliations:** ^1^ Key Laboratory of Adaptation and Evolution of Plateau Biota Northwest Institute of Plateau Biology & Institute of Sanjiangyuan National Park Chinese Academy of Sciences Xining China; ^2^ University of Chinese Academy of Sciences Beijing China; ^3^ Institute for Biology and Environmental Sciences Carl von Ossietzky‐University Oldenburg Oldenburg Germany; ^4^ Qinghai Provincial Key Laboratory of Crop Molecular Breeding Xining China

**Keywords:** chloroplast, *Comastoma*, Gentianaceae, phylogeny, plastome, Qinghai–Tibetan Plateau

## Abstract

Genus *Comastoma* (subt. Swertiinae, Gentianaceae) contains species, such as “Zangyinchen,” that are important herbs in Tibetan medicine. The phylogenetic relationship of this within Gentianaceae and the circumscriptions of its species have long been controversial with conflicting morphological and molecular data reported. Here, we used whole chloroplast genome sequences for *Comastoma* species and related taxa to reconstruct their phylogeny and clarify their taxonomic relationships. The results revealed that the length of all plastome sequenced varied from 149 to 151 kb and have high similarity in structure and gene content. Phylogenomic analysis showed that *Comastoma* is a monophyletic group, closely related to the genus *Lomatogonium*. The divergence time estimation showed that Gentianaceae diverged at about 21.81 Ma, while the split of *Comastoma* occurred at 7.70 Ma. However, the results suggested the crown age of species formation in this genus is after 4.19 Ma. Our results suggest that QTP uplift, the alternation of Quaternary glaciation and interglaciation, and monsoon changes might have acted as drivers of speciation in *Comastoma*.

## Graphical abstract

*Comastoma* is monophyletic and has the closest relationship with *Lomatogonium*. Gentianaceae diverged at about 21.81 Ma, while the split of *Comastoma* occurred at 7.70 Ma.

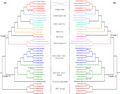

## 1 INTRODUCTION

As one of the most important global biodiversity hotspots, the Qinghai–Tibetan Plateau (QTP) and its adjacent regions harbor the world's richest recently diverged flora with high endemicity (Khan et al., 2018; Wu, 1988). The QTP is an important center of origin for many alpine taxa (Liu et al., 2002; Ren et al., 2015; Zhang et al., 2012) where lineages exhibit accelerated evolution as a consequence of the region's complex geological history (Muellner‐Riehl, 2019; Spicer, 2017). Gentianaceae is mainly distributed in cold temperate regions and comprises ~80 genera and ~700 species worldwide (Ho & James, 1995). As the biogeographical source area for several large alpine lineages, the QTP mountains host 22 genera and about 419 species (Ebersbach et al., 2017; Favre et al., 2016; Ho & Liu, 2001). Of these, the genus *Comastoma* (Gentianaceae) has about 15 species distributed in Asia, Europe, and North America. However, 11 of their species are only confined to the southwest and northwest of China. For local inhabitants in the QTP, *Comastoma* is one of the original plants of “Zangyinchen,” which are important traditional Tibetan medicine, widely used to treat hepatitis, liver fibrosis, and cholecystitis (Tang Li et al., 2008, 2020). *Comastoma* is named for the hairy bases of its corolla lobes, which has adaptive significance for reproductive success in the harsh environment of alpine regions (Zhang et al., 2018). While there have been many taxonomic and systematic treatments of Gentianaceae, the origin and phylogenetic position of *Comastoma* remains controversial (Hagen & Kadereit, 2002; Kissling et al., 2009; Schonswetter et al., 2004; Yuan & Kupfer, 1995). For example, Yuan and Kupfer (1995) divided the subtribe Gentianinae into two independent evolutionary branches as *Gentiana* and *Gentianella*, placing *Comastoma* in the second branch and suggested that *Comastoma* is monophyletic (Yuan & Kupfer, 1995). Furthermore, recent molecular phylogenetic surveys revealed *Comastoma*, *Lomatogonium*, and *Gentianella* are not monophyletic and are located in one more derived clades (Xi et al., 2014).

Further, results from Xi et al. showed *Lomatogonium* and *Gentianella* are on the same evolutionary branch which is corroborated by evidence that these genera can cross with each other (2014). Xi et al.'s (2014) results were parallel to Liu and Ho (1996), who hypothesized that *Comastoma* has the closest relationship with *Gentianella* based on the embryological characteristics (Xi et al., 2014). Toyokuni, however, considered *Comastoma* as a genus most closely related to *Lomatogonium* and distant from *Gentiana* and *Gentianella* (1961). This hypothesized relationship between *Comastoma* and *Lomatogonium* has been supported by molecular phylogenetic investigations (Chassot et al., 2001). Similarly, based on flower morphological characteristics, Wu et al. (2003) treated *Comastoma* as a separate genus and suggested that it is more primitive than *Gentianella* in phylogenetic position and started a new debate about its evolutionary history and position.

Most of the phylogenetic hypotheses about *Comastoma* evolution have been based on a few chloroplast markers as well as the ITS region and some morphological characters (Toyokuni, 1961; Wu et al., 2003; Xi et al., 2014). These molecular phylogenies used noncoding plastid regions with uniparental inheritance to infer the true species tree for groups with complex evolutionary histories (Hung et al., 2009; Valcárcel et al., 2003). Unfortunately, the most variable regions of the chloroplast genome were not known and even the most informative plastid regions did not have the resolving power of low‐copy nuclear markers (Shaw et al., 2007). To compensate for the comparably low variation in plastid genetic markers, whole plastome sequences are required to construct robust species trees (Hollingsworth et al., 2011; McCormack et al., 2013). Studies have proven that whole chloroplast genome sequencing can resolve phylogenetic relationships at various taxonomic levels and while also elucidating the molecular evolution of plastome structure and function (Jansen et al., 2007; Moore et al., 2007, 2010). Such valuable information has shown the effectiveness of full plastome data to resolve broader level questions at family, order, tribal, generic, and species levels (Barrett et al., 2016; Givnish et al., 2016).

Utilizing the high‐throughput sequencing technology and advanced statistical tools, we report for the first time a robust phylogeny and evolutionary history of genus *Comastoma* based on whole chloroplast sequences. The monophyly of *Comastoma* was tested in a phylogenetic context; if monophyletic, *Comastoma* species would form their cloud to the exclusion of species from all other genera. To this end, we sequenced the whole chloroplast genomes of five species of *Comastoma* and 11 other species representing four genera from Gentianaceae. Also, we included 19 complete chloroplast sequences from the NCBI: as *Swertia* (4 spps.), *Helenia conrniculata*, and *Gentiana* (14 spps.). The final aim is to (1) investigate the functional and structural differences in plastome of *Comastoma* and its allied taxa and (2) provide a robust phylogeny and evolutionary history of *Comastoma*.

## 2 MATERIALS AND METHODS

### 2.1 Species sampling

Fresh leaves from five *Comastoma* species, each represented with one individual, and 11 other species from the allied genera *Gentianella* (2 spps.), *Gentianopsis* (4 spps.), *Lomatogonium* (4 spps.), and *Tripterospermum volubile* were sampled from the QTP (Table 1). Of these, species included are those previously showed paraphyly in *Comastoma* (Xi et al., 2014). The leaves were silica gel dried during the fieldwork and kept at −20°C until total DNA extraction in the laboratory. Vouchers for all the samples are deposited into Qinghai–Tibetan Plateau Museum of Biology (HNWP), University of Chinese Academy of Sciences. Besides this, 19 species from Gentianaceae are included from the GenBank of NCBI: *Swertia* (4 spps.), *H. conrniculata*, and *Gentiana* (14 spps. Table 1). Species from Apocynaceae, Asclepiadaceae, and Rubiaceae were also downloaded from GenBank as outgroups.

**TABLE 1:** Sample information of *Comastoma* and its allied taxa

Species	Voucher No.	Location	Longitude	Latitude	GenBank Accession No.
*Comastoma pedunculatum*	chensl0546	Nangqian, QH	97°39′	34°07′	MN627282
*Comastoma pulmonarium*	chensl0683	Nangqian, QH	96°36′	32°18′	MN627286
*Comastoma polycladum*	chensl0726	Zaduo, QH	95°52′	32°56′	MN627288
*Comastoma jigzhiense*	chensl0430	Jiuzhi, QH	101°14′	33°24′	MN627283
*Comastoma falcatum*	chensl0120	Banma, QH	99°02′	34°49′	MK331815
*Gentianella azurea*	zhang2016495	Nangqian, QH	96°36′	32°13′	MN627289
*Gentianella arenaria*	zhang2018070	Zeku, QH	101°33′	35°04′	MN627277
*Tripterospermum volubile*	zhang20120123	Bomi, XZ	95°43′	29°37′	MN627287
*Gentianopsis barbata*	chensl0629	Shiqu, SC	97°21′	32°53′	MN627280
*Gentianopsis barbata* var. *stenocalyx*	chensl0691	Nangqian, QH	96°34′	31°52′	MN627276
*Gentianopsis paludosa*	chensl0762	Zaduo, QH	95°27′	32°51′	MN627278
*Gentianopsis paludosa* var. *aipina*	chensl0448	Aba, SC	102°05′	32°44′	MN627279
*Lomatogonium carinthiacum*	chensl0722	Zaduo, QH	96°01′	32°57′	MN627284
*Lomatogonium gamosepalum*	chensl0810	Qumalai, QH	95°50′′	34°01′	MN627285
*Lomatogonium perenne*	chensl0383	Banma, QH	100°35′	32°49′	MN627290
*Lomatogonium macranthum*	chen2014554	Yushu, QH	97°12′	32°34′	MN627281
*Swertia verticillifolia*	—	—	—	—	MF795137
*Swertia mussotii*	—	—	—	—	NC031155
*Swertia hispidicalyx*	—	—	—	—	NC044474
*Swertia bimaculata*	—	—	—	—	MH394372
*Halenia corniculata*	—	—	—	—	NC042674
*Gentiana veitchiorum*	—	—	—	—	NC037985
*Gentiana tibetica*	—	—	—	—	NC030319
*Gentiana straminea*	—	—	—	—	NC027441
*Gentiana stipitata*	—	—	—	—	NC037984
*Gentiana siphonantha*	—	—	—	—	NC039573
*Gentiana robusta*	—	—	—	—	KT159969
*Gentiana ornate*	—	—	—	—	NC037983
*Gentiana oreodoxa*	—	—	—	—	NC037982
*Gentiana officinalis*	—	—	—	—	NC039574
*Gentiana obconica*	—	—	—	—	NC037981
*Gentiana hexaphylla*	—	—	—	—	NC037980
*Gentiana dahurica*	—	—	—	—	MH261259
*Gentiana caelestis*	—	—	—	—	MG192304
*Gentiana crassicaulis*	—	—	—	—	KY595457
*Calotropis procera*	—	—	—	—	NC041440
*Carissa macrocarpa*	—	—	—	—	NC033354
*Dunnia sinensis*	—	—	—	—	NC039965
*Mitragyna speciosa*	—	—	—	—	NC034698
*Emmenopterys henryi*	—	—	—	—	NC036300

Note

QH, Qinghai Province, P.R. China; XZ, Xizang Autonomous Regions, P.R. China; SC, Sichuan Province, P.R. China.

### 2.2 DNA extraction, sequencing, and bioinformatics

Total genomic DNA was extracted following a modified CTAB protocol (Englen & Kelley, 2000) and was used to prepare Illumina sequencing libraries as described Thomson et al. (2018) and processed on an Illumina NovaSeq 6000 platform (Novogene) with 150 PE chemistry.

About 10 Gb raw data were obtained for each sample and processed with FastQC (http://www.bioinformatics.babraham.ac.uk/projects/fastqc/) to quality control; ~9 Gb clean reads were retained by Fastp (Chen et al., 2018). The reads were extracted by BWA v0.7.17 (Li & Durbin, 2009) and BLASTed against a reference plastome using BLAST v2.2.25 (Kent, 2002) and assembled by SPAdes v3.15.1 (Bankevich et al., 2012).

The final plastome was annotated by Geseq modular from CHLOROBOX (https://chlorobox.mpimp‐golm.mpg.de/geseq.html) and draw support from GENEIOUS (Matthew et al., 2012). We used the online module OGDRAW (https://chlorobox.mpimp‐golm.mpg.de/OGDraw.html) to drawn the circular diagram of plastome. Nucleotide variation and important characteristics (number of genes, genes content, gene loss, and IR border regions) were analyzed using DNASP v5.0 (Librado & Rozas, 2009). All chloroplast genome sequences obtained in this study were submitted to NCBI and assigned GenBank accession numbers (Table 1).

### 2.3 Chloroplast phylogenomics and diversification analyses

All sequence alignments were performed with MAFFT v7.471 (Katoh et al., 2019). Two different datasets were used to reconstruct *Comastoma* phylogeny: The first one included the complete genome of chloroplast while the second dataset included only protein‐coding sequences. Both the datasets included 35 ingroup and five outgroup species (details below) and were analyzed with maximum likelihood (ML) with IQ‐TREE v1.610 (Nguyen et al., 2014) and Bayesian inferences (BI) statistics MRBAYES v.3.2 (Ronquist & Huelsenbeck, 2003). Optimal substitution models were assessed with jMODELTEST using Akaike information criterion (Nguyen et al., 2014; Posada & Buckley, 2004). The ML analysis was performed using the GTR+F+R3 model and 1000 bootstrap replicates. The BI was conducted with two parallel runs of one million MCMCs, sampling after 1000 generations. A consensus tree was estimated after the first 25% of trees were discarded as burn‐in. In both ML/BI approaches, the trees were rooted with the outgroups including *Calotropis procera* (Asclepiadaceae), *Carissa macrocarpa* (Apocynaceae), *Mitragyna speciose*, *Dunnia sinensis*, and *Emmenopterys henryi* (Rubiaceae).

To assess the evolutionary history, we calibrated the divergence time of *Comastoma* and its related groups as implemented in BEAST (Drummond & Rambaut, 2007). For this analysis, we only used the whole chloroplast dataset in a concatenated fashion setting GTR as a substitution model with lognormal relaxed clock (Thomas et al., 2007).

We used 45‐Ma‐old infructescence and fruit fossils recovered for genus *Emmenoptery* (Rubiaceae) and seed fossil of 5 Ma age of *Gentiana*. Divergence times have been calibrated with sect. *Cruciata* (Gentianaceae) as this is the most recent common ancestor of genus *Comastoma* (Favre et al., 2016; Pirie et al., 2015).

The analysis was followed for 50 million generations with three independent MCMC runs, taking samples after every 5000 generations. We used the program Tracer v1.7.1 (http://tree.bio.ed.ac.uk/software/tracer/) to ensure that the ESS (effective sampling size) of each parameter was more than 200. After all parameters converged to exceed 200 ESS, we used Logcombiner v2.5.2 (https://www.onlinedown.net/soft/84600.htm) to combine all three runs. Finally, we used the program Tree Annotator v2.5.2 (Luo & Zhao, 2011) to generate the tree after burning the first 20% MCMC. The trees were displayed, annotated, and saved with the help of Figtree v1.4.0 (http://tree.bio.ed.ac.uk/software/figtree/).

## 3 RESULTS

### 3.1 Plastome comparison

**FIGURE 1:**
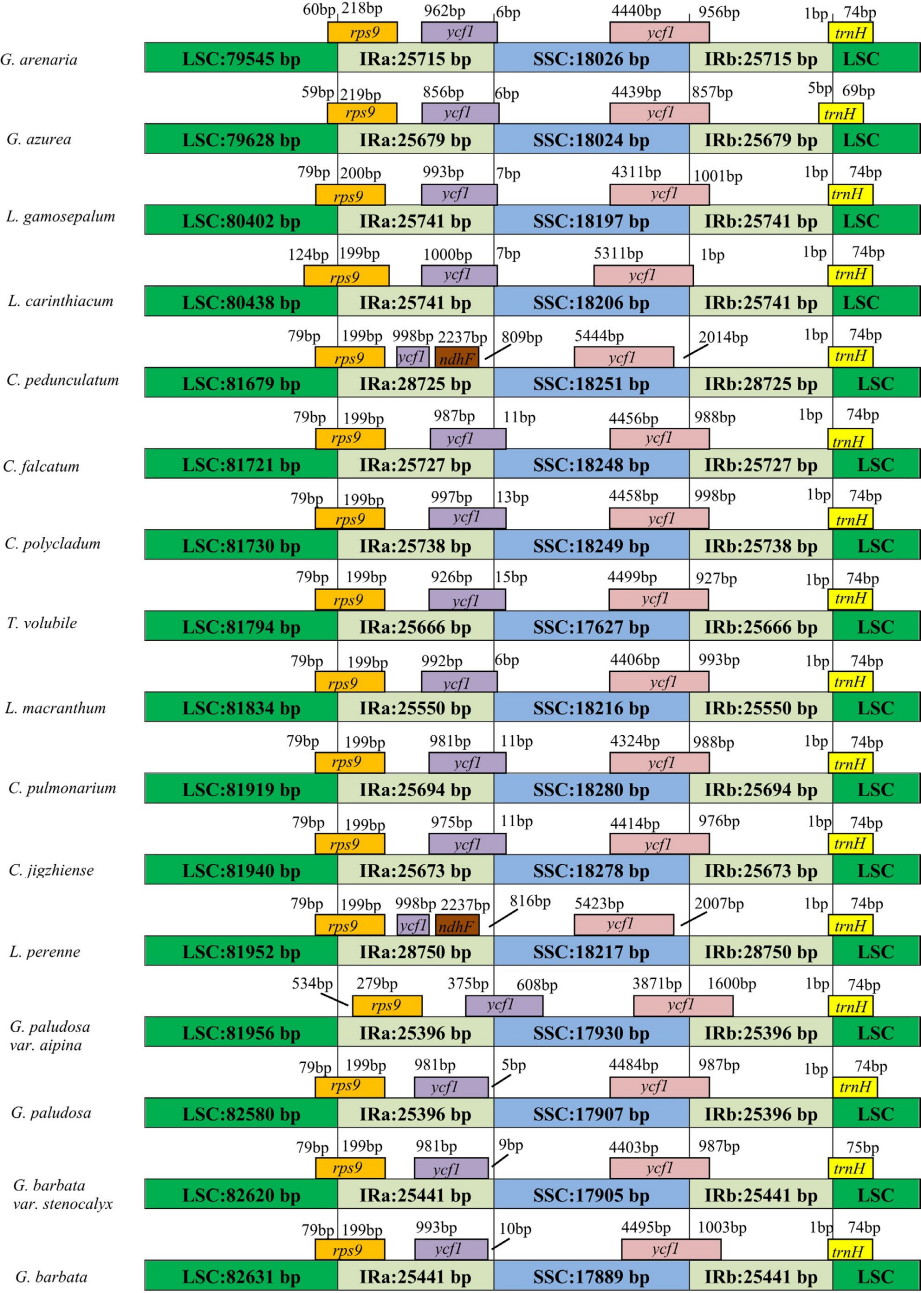
Showing comparison of junctions between the quadripartite structure in the LSC, SSC, and IR regions among 16 species. Distance in the figure is not to scale

**FIGURE 2:**
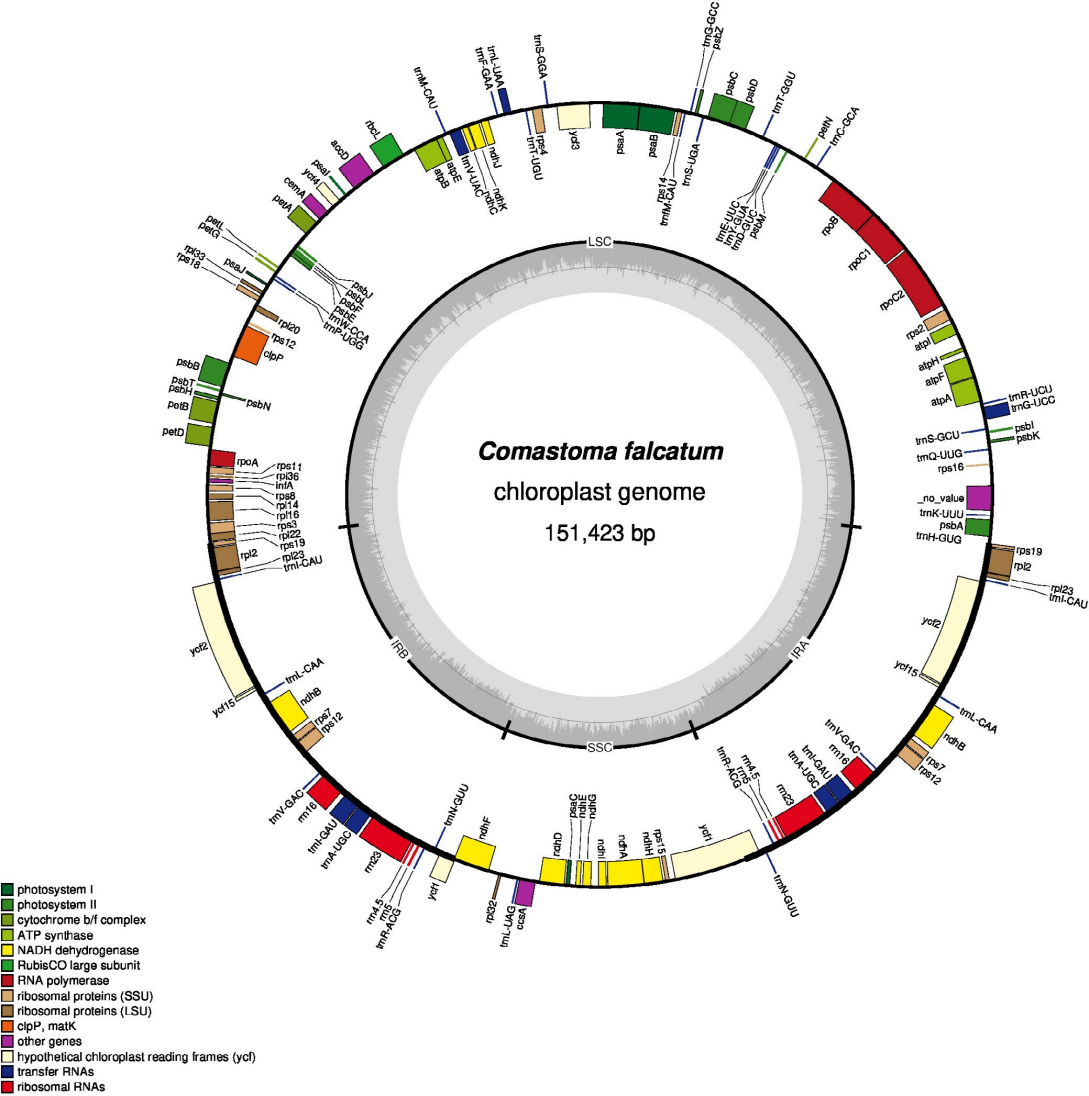
Plastome structure of *Comastoma falcatum*

**FIGURE 3:**
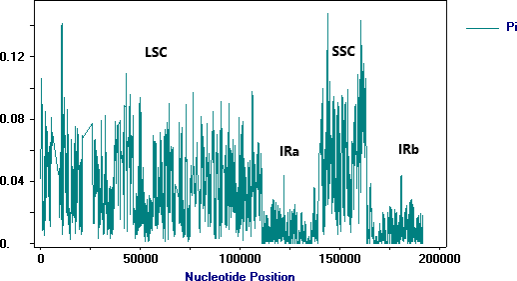
Nucleotide variation in chloroplast genomes of *Comastoma* and its allied taxa

All 16 sequenced chloroplast genomes displayed a typical quadripartite structure, including a large single‐copy region (LSC), a small single‐copy region (SSC), and two reverse repeat regions (IRa and IRb; Figure 1; Figure 2). Plastome size varied from 149,001 to 151,699 bp. The species belongs to the genus *Comastoma* possess 134 genes, including 89 protein‐coding genes (PCGs), 37 tRNA, and 8 rRNA genes. The details of protein‐coding genes were available in Appendix S1. The number of PCGs in other species ranged from 131 to 133, where the gene content has very subtle variation. We found the highest GC contents (38.27%) in *Comastoma polycladum* and the lowest (37.65%) in *T. volubile* (Table 2). We also found gene loss in other species, for example, *rps*16 has been lost in nine species (except *Comastoma*) and *rpl*33 in three species (*Gentianella azurea*, *Gentianella arenaria*, and *T. volubile*). Similarly, the number of rRNAs is the same in all species; however, the tRNAs are slightly different, for example, *trnS* ^GCU^ was lost in *Gentianopsis paludosa* and *Gentianopsis barbata*; and *trnV* ^UAC^ was lost in *T. volubile*. Our results found that the variation of plastome length is mainly due to the expansion and extraction of LSC and SSC regions. The IR regions were relatively conserved (Figure 3).

**TABLE 2:** Plastome characteristics of *Comastoma* and its allied taxa (Gentianaceae)

Species	Size (bp)				Number				GC content (%)
	Plastome	LSC	SSC	IR	Gene	PCGs	tRNA	rRNA	
*Comastoma pedunculatum*	151,380	81,679	18,251	28,725	134	89	37	8	38.26
*Comastoma pulmonarium*	151,587	81,919	18,280	25,694	134	89	37	8	38.25
*Comastoma polycladum*	151,455	81,730	18,249	25,738	134	89	37	8	38.27
*Comastoma jigzhiense*	151,564	81,940	18,278	25,673	134	89	37	8	38.26
*Comastoma falcatum*	151,423	81,721	18,248	25,727	134	89	37	8	38.26
*Gentianella azurea*	149,010	79,628	18,024	25,679	132	87	37	8	38.25
*Gentianella arenaria*	149,001	79,544	18,025	25,716	132	87	37	8	38.25
*Tripterospermum volubile*	150,753	81,794	17,627	25,666	131	87	36	8	37.65
*Gentianopsis barbata*	151,402	82,631	17,889	25,441	132	88	36	8	37.82
*Gentianopsis barbata var. stenocalyx*	151,407	82,620	17,905	25,441	133	88	37	8	37.86
*Gentianopsis paludosa*	151,279	82,580	17,907	25,396	132	88	36	8	37.86
*Gentianopsis paludosa var. aipina*	151,291	82,569	17,930	25,396	133	88	37	8	37.87
*Lomatogonium carinthiacum*	150,126	80,438	18,206	25,741	133	88	37	8	38.24
*Lomatogonium gamosepalum*	150,081	80,402	18,197	25,741	133	88	37	8	38.24
*Lomatogonium perenne*	151,699	81,952	18,217	28,750	133	88	37	8	38.16
*Lomatogonium macranthum*	151,150	81,834	18,216	25,550	133	88	37	8	38.13

### 3.2 Phylogenomics and diversification time

**FIGURE 4:**
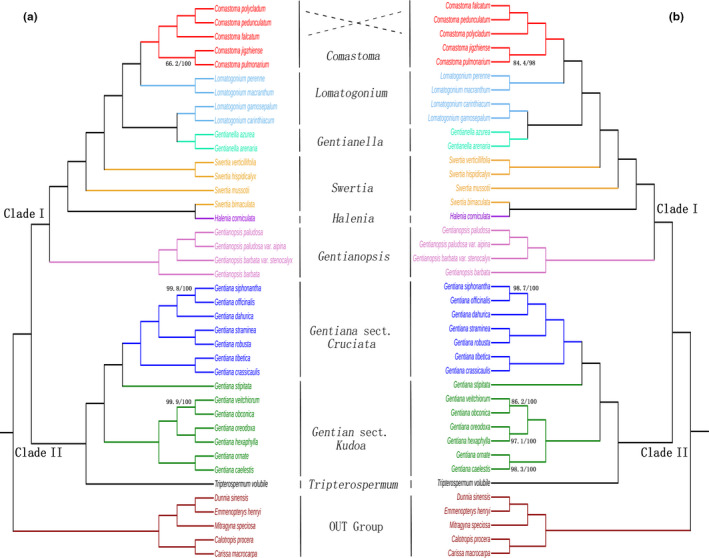
Phylogeny of *Comastoma* based on the maximum likelihood and Bayesian inferred trees (a) ML/BI tree based on complete chloroplast genomes of a concatenated data matrix. (b) ML/BI tree including only the protein‐coding sequences. A support rate less than 100 has been shown on the branches, where the first value represents the support rate of ML‐based statistics and the second value that of BI inferences

Both the ML and BI statistics recovered trees with the same topologies based on the whole chloroplast genome. Similarly, we recovered congruent trees based on only the protein‐coding sequence data. In all results, *Comastoma* species formed a clade sister to a couple of *Lomatogonium* species; together these taxa were sister to a clade containing other *Lomatogonium* species with *Gentianella* (Figure 4). The basal node of this clade was highly supported in both ML and BI reconstructions, suggesting *Comastoma* shares a common ancestor with genus *Lomatogonium*. Depending on the subset of data used, there were subtle inconsistencies in support for some branches and conflicting relationships among *Comastoma* species. In the ML/BI trees based on the whole chloroplast genome sequence, *Comastoma pedunculatum* has the closest relationship with *C. polycladum*, and then, these two species clustered together with *Comastoma falcatum*, while *C. pedunculatum* first aggregated with *C. falcatum* and then *C. polycladum* in the ML/BI trees using the protein‐coding sequence. The robustness of the phylogenies of each branch is relatively high above 90%.

**FIGURE 5:**
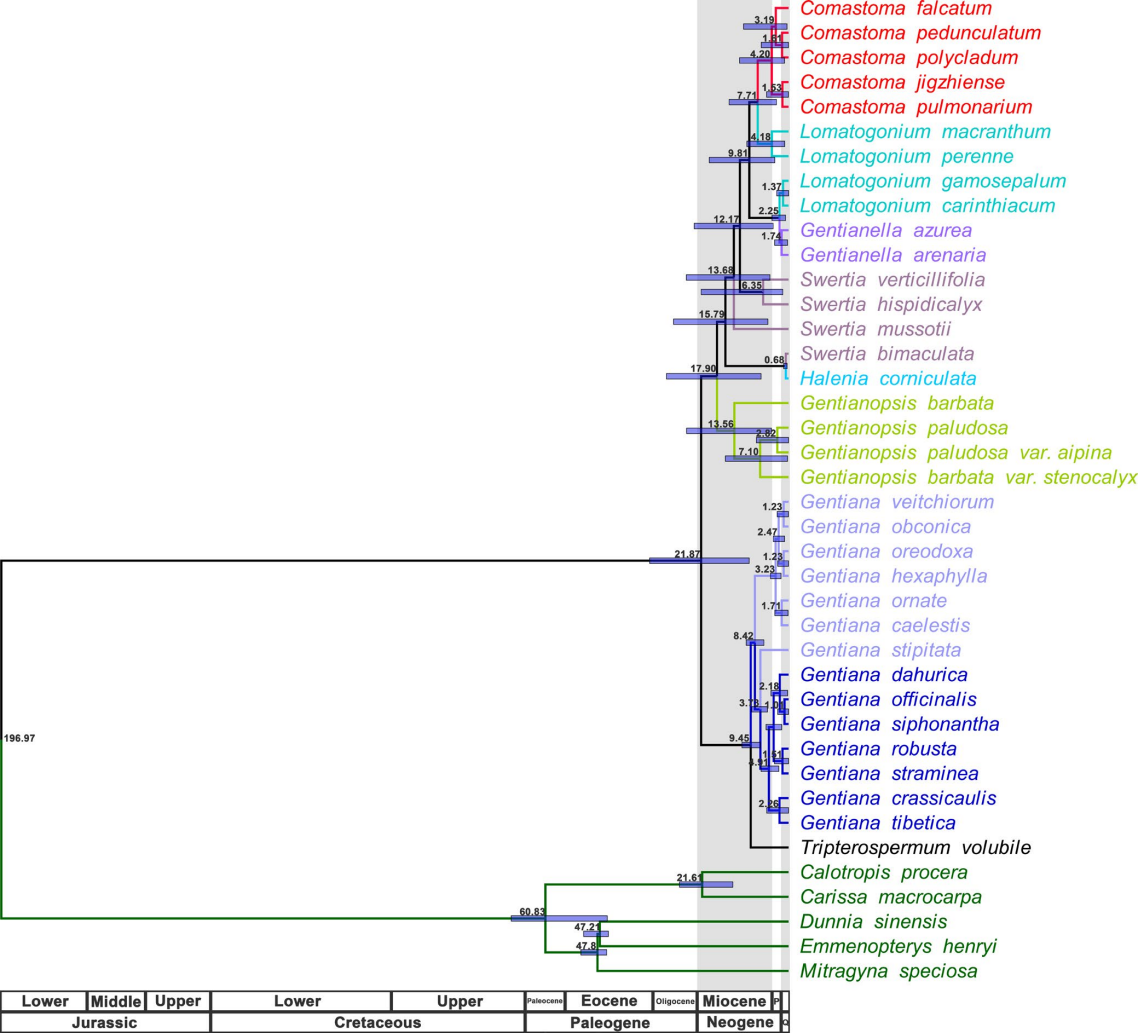
Molecular dating based on the complete chloroplast genome sequence. “P” represents Pliocene, “Q” represents Quaternary

The genus *Gentianopsis* is the most basal lineage followed by *Halenia corniculata* and then *Swertia*. The relationship among the four species in *Swertia* is relatively complex, for example, *Swertia bimaculata* has the closest relationship with *H. corniculata* rather than with another three species in *Swertia*. All the 14 species of *Gentiana* clustered in the same lineage with the species *T. volubile* (Figure 4). Time to the most recent common ancestor for all the 35 species of *Gentianaceae* included coalesced at 21.81 Ma (Figure 5). The most basal genus *Gentianopsis* diverged at about 17.89 Ma, followed by *Swertia*, *Halenia*, *Gentianella*, *Lomatogonium*, and *Comastoma*. The genus *Comastoma* diverged from its allied genus *Lomatogonium* at 7.70 Ma and is the most recently evolved group. Also, the genus *Comasatoma* further diverged into two subgroups divided the five species into two groups as *Comasatoma jigzhiense* and *Comasatoma pulmonarium*; and *C. pedunculatum* and *C. polycladum* and *C. falcatum*. The split between both the *Comastoma* species occurred about 4.19 Ma. Similarly, *T. volubile* diverged from *Gentiana* lineage at 9.45 Ma.

## 4 DISCUSSION

### 4.1 Plastome comparison

Genome structure, gene order, and gene content were highly conserved in five species of genus *Comastoma*. While sister groups (*Lomatogonium*, *Swertia*, *Tripterospermum*, and *Gentianella*) of *Comastoma* had similar plastome structures, there were distinctions among *Comastoma* and its close relatives, such as gene number and the *rps16* pseudogene. According to different studies (Oxelman et al., 1996; Roy et al., 2010; Wallander & Albert, 2000; Wanntorp & Källersjö, 2002), the *rps*16 is in an intergenic region or intron noncoding region, where the evolution rate is much faster than most of the genes in the chloroplast. Therefore, we suggest that this region can be used in the systematic evolution and the kinship investigation of Gentianaceae. Similarly, *rpl*33 was lost from *G. azurea*, *G. arenaria*, and *T. volubile*. This phenomenon might be based on two reasons: (1) *rpl*33 transferred from chloroplast genome to nuclear genome and (2) this gene is functionally nonessential. According to relevant research on *rpl*33, this gene could maintain enough plastid translation ability in cold environments (Rogalski et al., 2008). Most of the species in Gentianaceae grow in alpine areas with an average altitude of 4000 m, where the climate is relatively cold throughout the year.

In the light of our results and previous studies, *rpl*33 exists in most of Gentianaceae members with wide distribution and large numbers of species. In contrast, *rpl33* is not as common in less specious, like *Gentianella* and *Tripterospermum*. Therefore, the gene rpl33 may confer greater tolerance of cold climates; this explains the relatively smaller number of *Gentianella* and *Tripterospermum* on QTP. Similarly, in a study of the Tibetan herbs *Swertia hispidicalyx*, *Gentiana lhassica*, and *Halenia elliptica*, *trnS* ^GCU^ sequencing was suggested to be the best strategy for genetic diversity analysis and molecular identification (Ni et al., 2015). In our study, we found the loss of *trnS* ^GCU^ in *G. paludosa* and *G. barbata* plastomes, but not *G. paludosa var*. *aipina* and *G. barbata var*. *stenocalyx*. We can explore more about the importance of *trnS* ^GCU^ by including more individuals to assist morphology‐based distinction between varieties and species in *Gentianopsis*.

### 4.2 Phylogenomics of *Comastoma*

We found phylogenies based on whole chloroplast sequence data was more consistent and had higher bootstrap support values than using only CDS data. However, all the results showed *Comastoma* as one monophyletic group. Our result supports previous studies based on ITS sequences and embryological characteristics (Liu & Ho, 1996; Yuan & Kupfer, 1995). Our results are in contrast with Xi et al.'s results (Xi et al., 2014), where they found *Comastoma* as polyphyletic. In (Xi et al., 2014), ITS‐based phylogeny showed *C. pedunculatum* in one cluster but *matK* phylogeny recovered *C. pedunculatum* and *C. polycladum* with other groups as polyphyletic.

Despite our results substantiated the initial hypothesis that the genus is monophyletic by clustering all their five species, the interspecific relationship of species within the genus *Comastoma* showed inconsistencies based on different datasets. For example, *C. pedunculatum*, *C. falcatum*, and *C. polycladum* from a clade, but it is not clear whether *C. falcatum* or *C. polycladum* clustered with *C. pedunculatum* to the exclusion of the other. This distinction can be explained for two reasons. Firstly, evolutionary rates of coding and noncoding sequences vary and may contribute to discordant topologies (Tian & Li, 2002). Although some species *Lomatogonium* and *Swertia* for a clade in this study, more complete sampling of these genera are needed to fully understand the polyphyly of these genera. Secondly, we have included only one individual per species, which might lead to the inconsistency of results. This study supports that *Comastoma* is monophyletic, but there are not enough *Comastoma* species in this study, the clear phylogeny of *Comastoma* and Gentiana needs more in‐depth study in the future.

The overall phylogeny of different groups of Gentianaceae revealed *Comastoma* clustering with two species in *Lomatogonium* (*L*. *perenne* and *L*. *macranthum*) in a more recently evolved branch. Four species in *Gentianopsis* diverged earlier, so they have a more distant relationship with *Comastoma* than *Lomatogonium*. Interestingly, *L*. *gamosepalum* and *L*. *carinthiacum* clustered together with two species of *Gentianella* in the ML/BI tree (Figure 4a,b). Similarly, four species of *Swertia* not clustered in one group, for example, *S. bimaculata* has a closer sister relationship with *H. corniculata* rather than with the other three *Swertia* species. Although some species in *Lomatogonium* and *Swertia* for a clade in this study, more complete sampling of these genera is needed to fully understand the polyphyly of these genera. The 14 species of Gentiana formed a well‐supported clade. The overall topology of sect. *Kudoa* and sect. *Cruciata* is almost consistent with Sun's (2018) and Zhou's (2018) results. Support values for our topology were greater than those previously published (Sun et al., 2018; Xi et al., 2014; Zhou et al., 2018) because our whole plastome dataset includes vastly more phylogenetically informative characters.

### 4.3 Calibrated divergence time of *Comastoma*

The origin, distribution, and differentiation of many species have a close association with the change in the geology of the QTP (Ebersbach et al., 2017; Ren et al., 2015; Wang et al., 2009). *Comastoma* and its related groups are mainly distributed on the QTP. The uplift of the QTP is mainly divided into three stages. Based on the molecular clock hypothesis, the differentiation time of *Gentianaceae* has been estimated to be about 21.87 Ma and the divergence time between *Comastoma* and its related groups about 7.71 Ma. This time of differentiation is close to that of tectonic change of the QTP (Mosbrugger et al., 2018; Su et al., 2019; Xing & Ree, 2017). The subfamily differentiation of Gentianaceae and the emergence of *Comastoma* should be mainly influenced by the second and third stages of QTP uplifting. The second stage of QTP uplift occurred in 21–17 Ma, mainly in the southern part of the QTP and Himalayas, while the most massive uplift of the Tibetan Plateau occurred at 10–8 Ma, or more recently, that is after the Miocene (Spicer, 2017; Xing & Ree, 2017). With its rich and diverse ecosystem, the QTP has become a natural place for the convergence and fusion of various biota. In this area, there are multiple biological types and complex floristic components, which are ecosystems that are sensitive to climate change, but it is also the diversification hotspot of many natural populations and the cradle of speciation (Mosbrugger et al., 2018; Muellner‐Riehl, 2019). At 21–17 Ma, the rapid uplift of the QTP dramatically changed the surface pattern of the region, such as the emergence or disappearance of mountains, and river diversion. The complex geographical changes promoted the division of Gentianaceae into different genera in different directions after 21.87 Ma. Under the influence of long‐term geological and structural events, the climatic conditions for the survival of Gentianaceae plants also gradually changed. In the third stage of uplift, the barriers or channels of species diffusion were further accelerated, and the ecological and environmental gradients were expanded, which provided new niches for many plants of Gentianaceae, thus driving the differentiation of *Comastoma* and *Lomatogonium* in 7.71 Ma. Similarly, *Gentiana* appeared 9.45 million years ago, and then, subgenus differentiation occurred at 8.42 Ma, forming most of the species in the sect. *Kudoa* but except *Gentiana stipitata*. Then, in 7.03 Ma, the differentiation of sect. *Cruciata* happened. It shows that the emergence and rapid radiation of this genus are also obviously affected by the third stage of uplift in the QTP.

*Comastoma* subgenera diverged after 4.19 Ma, from the middle‐late Pliocene to early Quaternary. In this period, the subgenus differentiation of *Comastoma* is closely related to the influence of previous plateau uplifts (especially the third stage of uplift), but the role of Quaternary glaciers should not be ignored. In the Quaternary, the glacial and interglacial periods alternated periodically (Qiu et al., 2011). Climate changes that fluctuated with the glacial cycles contributed to the complex geographical environment on the QTP. The new niches and geographic isolation promoted the establishment of extraneous species from neighboring regions. At the same time, some species hybridized to produce new species. These factors led to the rapid radiation of the *Comastoma*. Changes in the monsoon cycle also presented new selective pressures asynchronous with the QTP uplift (Clift et al., 2008; Ji et al., 2005; Tada et al., 2016); such monsoon effects are often overlooked in biogeographic studies of this region. Therefore, we believe that the transformation of the monsoon climate may also be another essential driving force for the evolution of *Comastoma* and allied taxa.

## 5 CONCLUSIONS

The whole chloroplast genomes of *Comastoma* and its related taxa were sequenced for the first time to establish a robust hypothesis about the monophyly of this genus and its evolutionary relationships with other Gentianaceae. Analyzing our plastomes with 19 others from NCBI revealed the similarity of structure and content for our 16 species, underscoring the recent diversification of this lineage. Our results suggested that *Comastoma* is monophyletic and has the closest relationship with *Lomatogonium* through both ML and BI tree reconstruction. We further concluded that combined with the relevant geological and historical events, the uplift of the QTP, the alternation of Quaternary glaciation and interglaciation, and the change of monsoon may have created conditions inductive for speciation in the common ancestors of extant *Comastoma*. Additionally, we found that the whole chloroplast genome sequencing is an excellent strategy to better resolve the phylogeny of historically enigmatic groups at deep and recent divergences. We have substantiated the hypothesis of monophyly in *Comastoma* and suggest that such types of studies will provide helpful insight into solving similar problems in other groups.

## AUTHOR CONTRIBUTIONS

**Yu Zhang:** Data curation (equal); Formal analysis (equal); Investigation (equal); Visualization (equal); Writing‐original draft (lead). **Jingya Yu:** Data curation (equal); Investigation (equal); Visualization (equal); Writing‐review & editing (lead). **Mingze Xia:** Formal analysis (equal); Investigation (equal); Visualization (equal); Writing‐original draft (equal); Writing‐review & editing (equal). **Xiaofeng Chi:** Data curation (equal); Investigation (equal). **Gulzar Khan:** Formal analysis (equal); Investigation (equal); Writing‐review & editing (supporting). **Shilong Chen:** Conceptualization (lead); Data curation (equal); Investigation (equal); Resources (equal); Supervision (equal); Writing‐original draft (supporting). **Faqi Zhang:** Conceptualization (lead); Investigation (lead); Resources (supporting); Supervision (lead); Writing‐original draft (supporting); Writing‐review & editing (supporting).

## Supporting information

Appendix S1

## Data Availability

DNA sequences: GenBank accessions MN627276–MN627290, MK331815.
