# Supplementary material for: Plastome sequencing reveals phylogenetic relationships among Comastoma and related taxa (Gentianaceae) from the Qinghai–Tibetan Plateau
Source: Ecol Evol. 2021 Oct 28;11(22):16034–46. doi: 10.1002/ece3.8274 (PMC8601884; doi:10.1002/ece3.8274)
Supplement: Supplementary file 1 — Appendix S1 [file ECE3-11-16034-s001.docx]

Plastome sequencing reveals phylogenetic relationships among *Comastoma* and related taxa (Gentianaceae) from the Qinghai-Tibetan Plateau

APPENDIX 1

Yu Zhang ^1,2,a^, Jingya Yu ^1,2,a^, Mingze Xia ^1,2^, Xiaofeng Chi ^1^, Gulzar Khan ^3^, Shilong Chen ^1^ and Faqi Zhang ^1,4 *^

^1^ Key Laboratory of Adaptation and Evolution of Plateau Biota, Northwest Institute of Plateau Biology & Institute of Sanjiangyuan National Park, Chinese Academy of Sciences, Xining 810008, China

^2^University of Chinese Academy of Sciences, Beijing 100049, China

^3^ Institute for Biology and Environmental Sciences, Carl von Ossietzky-University Oldenburg, Oldenburg 26129, Germany

^4^ Qinghai Provincial Key Laboratory of Crop Molecular Breeding, Xining 810008, China

^a^ These authors contributed equally to this work.

⁎ Corresponding author at: Key Laboratory of Adaptation and Evolution of Plateau Biota, Northwest Institute of Plateau Biology & Institute of Sanjiangyuan National Park, Chinese Academy of Sciences, Xining 810008, China

Correspondence:

*Faqi Zhang*

*E-mail:* [fqzhang@nwipb.cas.cn](mailto:fqzhang@nwipb.cas.cn)

**Appendix S1:** Details of Protein-coding genes of *Comastoma falcatum.*

| Function | Gene Group | Gene Name |
| --- | --- | --- |
| Self-replication | Ribosomal RNA genes | *rrn16S, rrn23S, rrn4.5S, rrn5S* |
|  | Transfer RNA genes | *trnC*^GCA^*, trnD*^GUC^*, trnE*^UUC^*, trnF*^GAA^*, trnG*^GCC^*, trnH*^GUG^*, trnI*^CAU^*, trnL*^CAA^*, trnL*^UAG^*, trnM*^CAU^*, trnfM*^CAU^*, trnN*^GUU^*, trnP*^UGG^*, trnQ*^UUG^*, trnR*^ACG^*, trnR*^UCU^*, trnS*^GCU^*, trnS*^GGA^*, trnS*^UGA^*, trnT*^GGU^*, trnT*^UGU^*, trnV*^GAC^*, trnW*^CCA^*, trnY*^GUA^*, trnI*^GAU^*, trnA*^UGC^*, trnK*^UUU^*, trnG*^GCC^*, trnL*^UAA^*, trnV*^UAC^ |
|  | Small subunit of ribosome | *rps2, rps3, rps4, rps7, rps8, rps11, rps12, rps14, rps15, rps16, rps18, rps19* |
|  | Large subunit of ribosome | *rpl2, rpl14, rpl16, rpl20, rpl22, rpl23,rpl32, rpl33, rpl36* |
|  | DNA dependent RNA polymerase | *rpoA, rpoB, rpoC1, rpoC2* |
| Photosynthesis | Photosystem I | *psaA, psaB, psaC, psaI, psaJ* |
|  | Photosystem II | *psbA, psbB, psbC, psbD, psbE, psbF, psbH, psbI, psbJ, psbK, psbL, psbM, psbN, psbT, psbZ* |
|  | Cytochrome | *petA, petB, petD, petG, petL, petN* |
|  | ATP synthase | *atpA, atpB, atpE, atpF, atpH, atpI* |
|  | NADH dehydrogenase | *ndhA, ndhB, ndhC, ndhD, ndhE, ndhF, ndhG, ndhH, ndhI, ndhJ, ndhK* |
|  | Large subunit of Rubisco | *rbcL* |
|  | ATP-dependent protease subunit | *clpP* |
| Other functions | Maturase | *matK* |
|  | Envelope membrane protein | *cemA* |
|  | Translational initiation factor | *infA* |
|  | Subunit of acetyl-CoA-carboxylase | *accD* |
|  | C-type cytochrome synthesis gene | *ccsA* |
| Unknown function | Conserved open reading frames | *ycf1, ycf2, ycf3, ycf4, ycf15* |
